# Strong species structure but weak geographical structure in demersal Lake Victoria cichlids

**DOI:** 10.1002/ece3.9669

**Published:** 2022-12-25

**Authors:** Jacco C. van Rijssel, Florian N. Moser, Salome Mwaiko, Ole Seehausen

**Affiliations:** ^1^ Department of Fish Ecology & Evolution EAWAG Centre for Ecology, Evolution and Biogeochemistry Kastanienbaum Switzerland; ^2^ Institute of Ecology and Evolution, Aquatic Ecology University of Bern Bern Switzerland; ^3^ Wageningen Marine Research Wageningen University IJmuiden The Netherlands

**Keywords:** cichlids, haplochromines, hybridization, Lake Victoria, RAD‐tag sequencing, speciation, species delineation

## Abstract

Studying phenotypic and genetic differentiation between very young species can be very informative with regard to learning about processes of speciation. Identifying and characterizing genetic species structure and distinguishing it from spatial genetic structure within a species is a prerequisite for this and is often not given sufficient attention. Young radiations of cichlid fish are classical speciation study systems. However, it is only during the past decade that population genomics based on next‐generation sequencing has begun to provide the power to resolve species and distinguish speciation from spatial population structure for the youngest of these radiations. The Lake Victoria haplochromine cichlids constitute the youngest large cichlid fish radiation, probably <20,000 years old. Earlier work showed that communities of rocky reef cichlids are composed of many reciprocally monophyletic species despite their very recent origins. Here, we build on this work by studying assemblages of offshore demersal cichlids, adding analyses of within‐species spatial structure to the sympatric species structure. We sampled seven multispecies communities along a 6‐km‐long transect from one side of the Mwanza Gulf to the other side. We investigated whether phenotypically diagnosed putative species are reciprocally monophyletic and whether such monophyly is stable across species geographic ranges. We show that all species are genetically strongly differentiated in sympatry, that they are reciprocally monophyletic, and that monophyly is stable across distribution ranges. We found significant differentiation between geographically distinct populations in two species, but no or weak isolation by distance. We further found subtle but significant morphological differences between all species and a linear relationship between genomic and morphological distance which suggests that differences in morphology begin to accumulate after speciation has already affected genome‐wide restrictions of gene flow.

## Graphical abstract

The haplochromine cichlid fish of Lake Victoria, East Africa, are a recently diverged species flock where finding species structure with genetic data and reconstructing the phylogenetic relationships between species is a notoriously difficult problem due to its extraordinary diversity of >500 species. Using next‐generation sequencing, this is the first study showing monophyly in Lake Victoria cichlids across a relatively wide geographical range confirming that these very closely related species are real species and not locally adapted populations. The strong correlation between genomic and morphological divergence also suggests that divergent selection on morphology becomes most effective after speciation has already occurred.

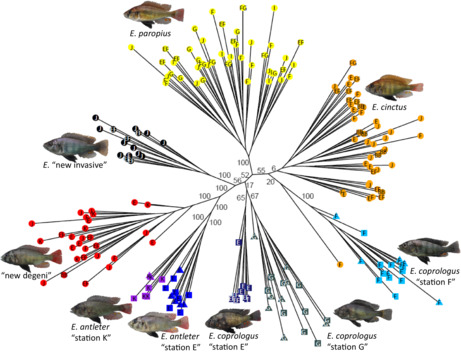

## 1 INTRODUCTION

Studying phenotypic and genetic differentiation between very young species can be informative with regard to learning about processes of speciation. Identifying and characterizing genetic species structure and distinguishing it from spatial genetic structure within a species is a prerequisite for this, which is often not given sufficient attention. Young radiations of cichlid fish are classical speciation study systems. However, it is only during the past decade that population genomics using next‐generation sequencing (NGS) has begun to provide the power to resolve young species and distinguish them from spatial population structure for the youngest of these radiations.

Identifying mechanisms of adaptation and speciation has been a major goal of evolutionary ecologists since the field emerged. Fish are often used as model taxa to identify these mechanisms. Ray‐finned fishes comprise about half of the diversity of all vertebrates, with 32,513 currently described species (Bánki et al., 2022). Remarkably, almost half of these occur in freshwater, while freshwaters only entail <1% of the Earth's surface. In contrast, the diversity of ray‐finned fishes in the oceans is roughly equal to that of freshwaters while oceans cover 70% of the Earth's surface (Levêque et al., 2007; Vega & Wiens, 2012). This enormous diversity of freshwater fishes has spiked the interest of many evolutionary ecologists trying to unravel the origin of species richness in clades. As a result, freshwater fish “species flocks”, often occurring as a textbook example of adaptive radiation, have increased our understanding of speciation in spatially confined ecosystems such as a single lake. The best‐studied cases of speciation in freshwater fishes have been investigated for many years from the perspectives of natural history, ecology, and genetics, and these data greatly aid our understanding of their evolutionary history (Seehausen & Wagner, 2014). Now, with the arrival of NGS, our knowledge of these systems is advancing rapidly. The use of these approaches is increasing in popularity in studies of adaptive radiations (Andrews et al., 2016; de la Harpe et al., 2017). The development of these approaches has made it possible to conduct phylogenomic studies of recently diverged taxa with increasing levels of taxon sampling within these groups. Especially studies on tropical fish taxa have taken advantage of the population genomic developments to delineate species boundaries, reconstruct phylogenetic relationships, and detect introgression (Alter et al., 2017; Ford et al., 2015; Franchini et al., 2017; Kautt et al., 2016; Keller et al., 2013; Machado‐Schiaffino et al., 2017; Martin et al., 2015, 2016; Martin & Feinstein, 2014; Meier, Marques, et al., 2017; Meier, Sousa, et al., 2017; Wagner et al., 2013). For some of the studied model organisms, restriction site‐associated DNA markers (RAD tags; Baird et al., 2008), were used to study population genomics of taxa for which microsatellites or AFLP studies already showed divergence between species (e.g., Kautt et al., 2012). Whereas others targeted recently diverged species that could not be separated with use of microsatellites or could not recover species structure in individual‐based trees, clustering, or assignment tests (e.g., Konijnendijk et al., 2011).

The haplochromine cichlid fish of Lake Victoria, East Africa, are such a species flock that finding species structure with genetic data and reconstructing the phylogenetic relationships between species is a notoriously difficult problem due to its extraordinary diversity of >500 species (Greenwood, 1974; Seehausen, 1996; Witte et al., 2007), extremely recent origin (<15,000 years; Johnson et al., 1996), and ancient (Meier, Marques, et al., 2017) as well as ongoing hybridization (Meier, Sousa, et al., 2017; Seehausen, van Alphen, & Witte, 1997) between species. Meier, Marques, et al. (2017) found evidence that the entire regional species flock (that also includes Lakes Kivu, Edward, and Albert) originated from hybridization between two distantly related species and that this might have facilitated the fastest, extensive, vertebrate radiation known. Shared polymorphism due to incomplete lineage sorting is expected to be high for these closely related species (Nagl et al., 1998), and some authors have used the presence of extensively shared genetic variation among morphologically and ecologically distinct sympatric species to question whether this is a species radiation (Samonte et al., 2007).

Pre‐genomics studies of closely related sympatric sister species generally showed small but significant *F*_ST_ values (mitochondrial sequences: Mzighani et al., 2010; microsatellites: Magalhaes et al., 2009; Magalhaes et al., 2012; Seehausen et al., 2008; AFLPs: Bezault et al., 2011; Konijnendijk et al., 2011). However, these studies were unable to recover species structure in individual‐based trees, PCA, or clustering. Using RADseq, Wagner et al. (2013) were the first to produce evidence for reciprocal species monophyly that supported morphological diagnoses of 16 sympatric species at one offshore rocky reef in Lake Victoria.

So far, no NGS approaches have been applied to the demersal haplochromines. Therefore, we studied the genomic sequence data from RAD loci to test species hypotheses and reconstruct phylogenetic relationships in demersal Lake Victoria cichlids. We investigated these using six putative species (based on their ecomorphology, for readability purposes, we use the term “species” from now on) from a well‐studied research transect in the Mwanza Gulf of southern Lake Victoria. This research transect has been monitored by the Haplochromis Ecology Survey Team (HEST) since the 1970s and this part of the Mwanza Gulf has endured severe environmental changes since the 1980s (van Rijssel et al., 2016) which resulted in a haplochromine species biodiversity loss of approximately 40% of both described as undescribed species (Witte et al., 1992). During the 1990s, some of these species recovered (Seehausen, Witte, et al., 1997; Witte et al., 2000).

## 2 MATERIAL AND METHODS

### 2.1 Study species

**FIGURE 1:**
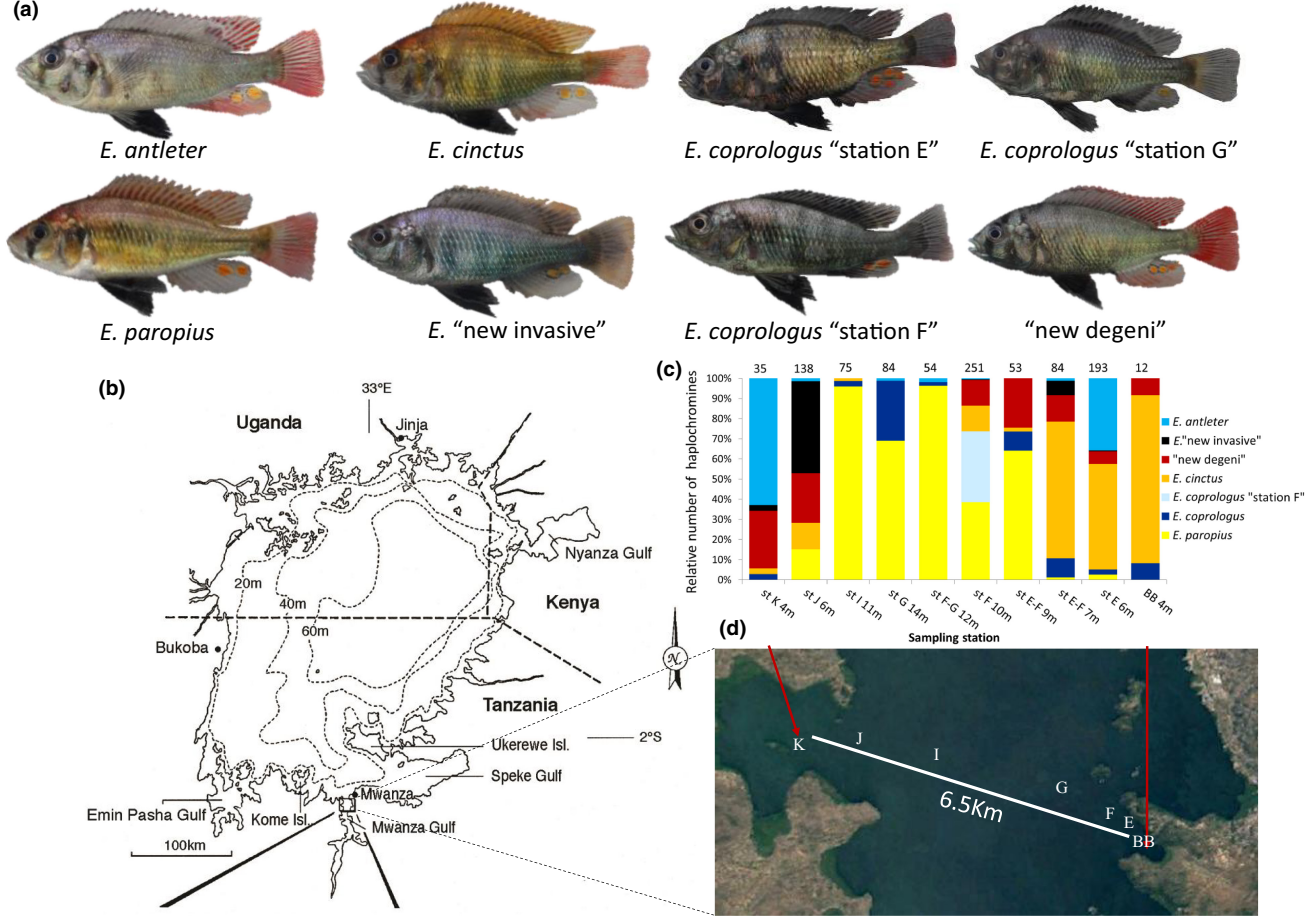
(a) Eight putative species studied on the (b) research transect of 6.5 km with sampling stations in the Mwanza Gulf of Lake Victoria and (c) the relative abundance of haplochromines on the research transect. The numbers above bars indicate the absolute number of haplochromines caught at each sampling station while numbers below the bars indicate the depth. Absolute numbers of *E. paropius* at the deeper stations (F, F‐G, G, I) are lower bound estimates as not all *E. paropius* specimens of each catch were counted and preserved. Sampling stations are: BB = Butimba Bay, E = station E, F = station F, G = station G, H = station H, I = station I, J = station J, and K = station K.

Six previously only phenotypically diagnosed (putative) species of demersal haplochromines were sampled by bottom trawling at seven sampling stations (Butimba Bay, E, F, G, I, J, K) on a 6‐km‐long research transect across the Mwanza Gulf in southern Lake Victoria, Tanzania, in 2014 (Figure 1). The sampling sites ranged from 4 to 14 m water depth and all had muddy bottoms. At least four of these putative species mainly feed on detritus and phytoplankton and one species used to feed on snails and detritus. Those species for which diet data had been published also incorporate other prey such as zooplankton, midge larvae, other insect larvae, shrimps, and mollusks, and increasingly so after the recovery from near extinction of all these species in the 1980s/90s (Kishe‐Machumu et al., 2008; van Rijssel et al., 2015). The detritivorous/phytoplanktivorous species (hereafter called detritivores) are the numerically most abundant trophic guild on the research transect (Kishe‐Machumu et al., 2015; Witte et al., 1992). Phenotypically, detritivores are distinguished from zooplanktivores based on their relative deep body (body depth > 30% standard length) and a less acute snout. All species occur in sympatry on the transect and most even occur syntopically (Figure 1c). All of these species differ in their distinctive male nuptial coloration (Figure 1a), whereas the females of all are very similarly brassy colored. Two of these species spurt two distinct male nuptial color morphs that we refer to as blue and red morphs (see below). Because we could not detect any other phenotypic difference between these, we hypothesized that these were color morphs within the same species.

The most abundant haplochromine on the research transect in these past 10 years has been the detritivore *Enterochromis paropius* Greenwood & Gee, 1969 (“paropius‐like” in Witte et al., 1992; Kishe‐Machumu et al., 2015; “broken bar” in Seehausen, Witte, et al., 1997). This species has a red dorsum and a bright yellow flank with a very distinct mid‐lateral band in both males and females and only faint if any vertical bars. It used to occur at 15–30 m water depth (Greenwood & Gee, 1969) and used to be rare on the shallower research transect before its near extinction (Witte et al., 1992).

The detritivorous *Enterochromis cinctus* Greenwood & Gee, 1969 has a red dorsum and a bright yellow flank too but it has dark vertical bars and lacks any mid‐lateral band. Before the near extinctions, it used to occur at depths between 13 and 60 m (Greenwood & Gee, 1969).

The detritivorous *Enterochromis antleter* Mietes & Witte 2010 (formerly known as *E*. “dusky wine red fin”; Witte et al., 1992) is dusky blue‐gray with faint vertical bars. Its caudal fin has a dusky base and is wine‐red otherwise. The species used to occur over mud bottoms in the sublittoral part of the Mwanza Gulf at 2‐11 m (Witte et al., 1992). This is one of the two species in which we found both blue and red morph males.

The detritivore *Enterochromis coprologus* Niemantsverdriet & Witte 2010 (formerly known as *E*. “nigrofasciatus”; Witte et al., 1992) has a nuptial coloration that is almost completely blue‐black while the dorsum, flank, and chest can have a silvery sheen (de Zeeuw et al., 2010). Next to detritus, diatoms and copepods made up a large part of its diet and it occurred in the sublittoral part of the Mwanza Gulf before the near extinctions (Goldschmidt et al., 1993; Witte et al., 1992). This is the other species in which we observed both blue and red morph males at stations F and G, but not at station E.

The fifth detritivorous species, *Enterochromis* sp. “new invasive”, is an undescribed species assumed to belong to the detritivores based on its morphology and habitat utilization. Males have a dark blue, almost black flank with faint vertical bars and a dusky dorsal fin with distinctively orange lappets. It is of smaller size, and has a slenderer body and more acute snout (somewhat reminiscent of the zooplanktivorous genus *Yssichromis*) than the other detritivores. It occurs above mud in the sublittoral areas of the Mwanza Gulf. This species was only discovered by one of us (OS) in the early 2000s but is now abundant and widespread (hence, its name “new invasive”).

Morphologically similar, the four first mentioned detritivorous species mainly differ in male coloration. *Enterochromis cinctus* and *E. paropius* are even considered morphologically indistinguishable by Greenwood and Gee (1969). In the species descriptions of de Zeeuw et al. (2010), *E. cinctus* and *E. antleter* were also considered morphologically similar. *Enterochromis coprologus* is the only described detritivore that differs notably in external morphology from *E. cinctus*, *E. antleter*, and *E. paropius* by its dorsal head profile, which is straight to incurved above the eye and moderately decurved in the other three detritivores (Goldschmidt et al., 1993). Also, the lower jaw in *E. coprologus* is longer, narrower, and more oblique than in these other three species (de Zeeuw et al., 2010).

The sixth species studied resembles the snail sheller *Platytaeniodus degeni* Boulenger, 1906 but is distinct from it genetically and phenotypically (Marques et al., in prep). *Platytaeniodus degeni* used to feed on snails and detritus and was last seen in 1991 (Seehausen, Witte, et al., 1997). The current taxon, which we refer to as “new degeni”, feeds on midge larvae, mollusks, and shrimps (van Rijssel et al., 2015). *Platytaeniodus degeni* had a very distinct, unique morphology with horseshoe‐shaped oral jaws that hold broad bands of small conical teeth and it used to be the only species of this genus. While “new degeni” is less extreme, its morphology remains unique and because of the relatively large proportion of snails in its diet (van Rijssel et al., 2015), we consider it more of a molluscivore than the other species in this study. The nuptial males of this species have a light blue body with iridescent blue lips. *Platytaeniodus degeni* used to occur over littoral sand and mud bottoms but the current taxon has a wider habitat that ranges from 4 to at least 10 m depth over sublittoral mud bottoms (Kishe‐Machumu et al., 2015; Witte et al., 1992).

Identification of individuals to species was done by OS based on standardized live fish photographs made in a custom‐made photo‐cuvette immediately after capture. After capture, fish were euthanized by an overdose of phenoxyethanol diluted in water (after which a fin clip was taken for genomic analyses) and were fixed in 4% formaldehyde. The fish were later transferred in three steps to 30%, 50%, and 75% ethanol and are stored at the EAWAG Centre for Ecology, Evolution and Biogeochemistry, Kastanienbaum, Switzerland.

### 2.2 Morphological measurements and analysis

We measured 15 morphological traits that have proven powerful for quantifying intraspecific and interspecific morphological variation in haplochromine cichlids of Lake Victoria (Barel et al., 1977): standard length (SL), head length (HL), head width (HW), body depth (BD), lower jaw length (LJL), lower jaw width (LJW), snout length (SnL), snout width (SnW), cheek depth (ChD), preorbital depth (POD), preorbital width (POW), interorbital width (IOW), eye length (EyL) and depth (EyD), and premaxillary pedicel length (PPL; Figure S1, Supplementary Material). We measured 661 individuals using digital calipers (Table S1, Supplementary Material). A linear discriminant function analysis (LDA) was used to visualize the morphological variation among the species and their color morphs. To test for morphological differentiation between species and morphs, we calculated the Bhattacharyya distance (Bd, which takes into account differences in standard deviations between clusters) between each species or color morph pair in the LD1‐LD2 space using the bhattacharyya.dist function from the R package “fpc” (R development Core Team, 2017). The significance of Bds was tested with Hotelling's *T*‐squared test. A sequential Bonferroni correction was used to adjust the *p*‐values.

### 2.3 DNA extraction and RADseq analysis

We chose 9 to 46 individuals of each species/population from different sampling stations for sequencing (Table S2), for a total of 198 individuals in the complete dataset. DNA was extracted from fin clips using a DNeasy Blood & Tissue kit (Qiagen) following the manufacturer's instructions. RADseq was performed following a standard protocol (Baird et al., 2008) with minor modifications described in Meier, Sousa, et al. (2017). Restriction digestion was performed overnight using the restriction endonuclease HF‐SbfI (NewEngland Biolabs) and 400 ng DNA per sample. P1 adapters contained 5‐ to 8‐bp‐long barcodes, each differing by at least two nucleotides from all other barcodes. The DNA was sheared with a Covaris S220 Focused‐Ultra sonicator, and fragments of 300–600 bp length were selected with a Sage Electrophoretic Lateral Fractionator (SageELF) instead of visual size selection on an agarose gel. All libraries were single‐end sequenced on an Illumina HiSeq 2500 sequencer.

The reads were demultiplexed and trimmed to 92 bp with the process_radtags script from the Stacks pipeline (Catchen et al., 2013), correcting single errors in the barcode and the restriction site, and discarding reads with incomplete restriction sites. The reads of each individual were then mapped to the *Pundamilia nyererei* reference genome (accession: GCF_000239375.1; Brawand et al., 2014) using Bowtie2 v. 2.2.6 (Langmead & Salzberg, 2012) with the end‐to‐end alignment option and default parameters. SAMtools v0.1.19 (Li et al., 2009) was used to convert alignments to binary format. We recalibrated base quality scores of aligned reads using empirical error rate estimations derived from bacteriophage PhiX reads following Marques et al. (2016). Raw sequencing reads from each lane were aligned against the PhiX 174 reference genome (accession: NC_001422; Sanger et al., 1977), known variation was masked and PhiX alignments were used to create a base quality score recalibration table for each lane and library combination using BaseRecalibrator from GATK v.2.7 (McKenna et al., 2010). Single‐nucleotide polymorphisms (SNPs) and genotypes were called using GATK Unified Genotyper v. 3.5 (McKenna et al., 2010). Afterward, all sites were filtered with a custom‐made Python script, BCFTOOLS v. 0.1.12, and VCFTOOLS v. 0.1.14 (Danecek et al., 2011). Genotypes were required to have a depth of coverage of at least 10 reads and a minimum quality value (GQ) of 30. Sites with more than 40% missing data were removed.

### 2.4 Population genomics

To study population structure among our species, we performed principal component analysis (PCA) with the R‐package SNPRelate (Zheng et al., 2012) using biallelic SNPs from the RAD‐sequencing dataset with a minor allele frequency of at least 5% overall sequenced individuals. We applied Mantel tests with 1000 permutations to assess whether genetic distance between populations was correlated with waterway distance between the stations using ARLEQUIN v. 3.5.2.3 (Excoffier & Lischer, 2010). The same software was used to calculate pairwise *F*_ST_ values to characterize levels and heterogeneity of genomic differentiation between species pairs; significance of these *F*_ST_ values was calculated with a permutation test (100 iterations). We performed Bayesian clustering assignment of all individuals with 1 to 10 clusters (*K* = 1–10) using STRUCTURE v. 2.34 (Pritchard et al., 2000). We ran 10 replicates each assuming 1 to 10 clusters with 100,000 burn‐in and 200,000 sampling steps, and checked convergence of replicates visually. The most likely number of clusters was identified by the highest delta K among all runs (Evanno et al., 2005) with STRUCTURE HARVESTER (Earl & Vonholdt, 2012). We used a maximum‐likelihood approach in RAxML 8.0.0 for phylogenetic analyses (Stamatakis, 2014). We used a GTR + gamma model of sequence evolution for single full‐ML tree searches and 100 replicates of RAxML's rapid bootstrap algorithm to account for uncertainty in the estimation of the topology (Stamatakis et al., 2008) following Wagner et al. (2013). We also tested for a correlation between genomic and morphological differentiation by correlating pairwise *F*_ST_‐values and Bhattacharyya distances (LD1‐LD2) using a Spearman correlation test after data were shown to be non‐parametric by a Shapiro–Wilk's test.

## 3 RESULTS

After alignment to the reference genome and genotype calling, we obtained a genotypic dataset of 1,975,077 bp. After all filtering steps, we retained 8609 SNPs with maximum of 40% missing data at a minor allele frequency level of 5% and a mean depth of 46× (range 11–182X, Figure S2).

### 3.1 Neither genetic nor morphological differences between conspecific color morphs

**FIGURE 2:**
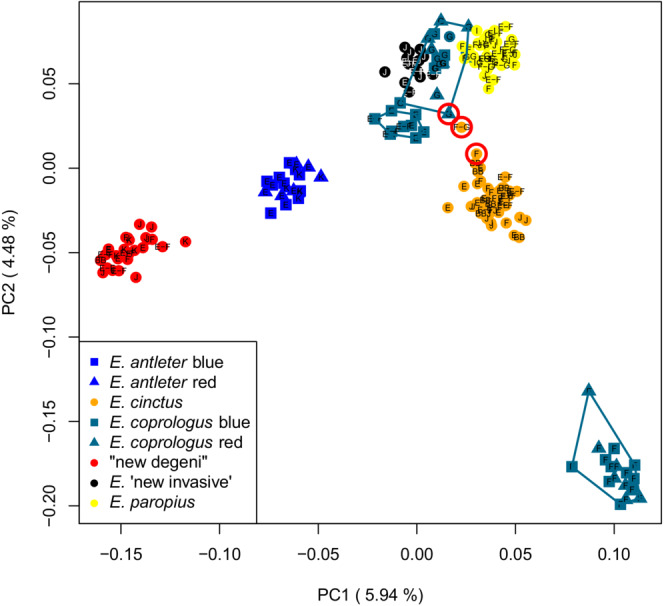
PCA plot showing the genetic differentiation between species based on 8609 SNPs. The first two axes are shown with the percentage of variance explained in parentheses. Different symbols represent individuals from different species as indicated in the legend. The red open circles indicate individuals that show the highest number of shared alleles according to the STRUCTURE analysis.

**FIGURE 3:**
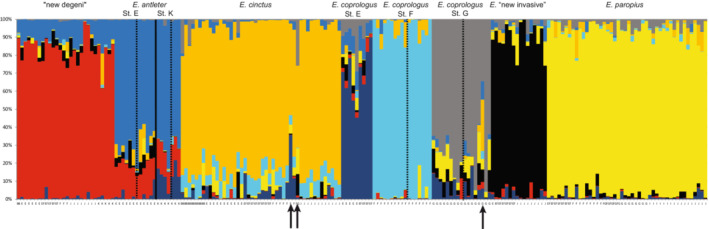
STRUCTURE analysis with eight clusters (*K* = 8) based on 8609 biallelic SNPs with minor allele frequency of at least 0.05. The vertical line indicates the split between *E. antleter* from station E and station K. The dotted vertical lines indicate the splits between blue (left) and red (right) morphs. Note the two *E. cinctus* and the one *E. coprologus* “station G” individuals that share a high number of alleles (black arrows).

**FIGURE 4:**
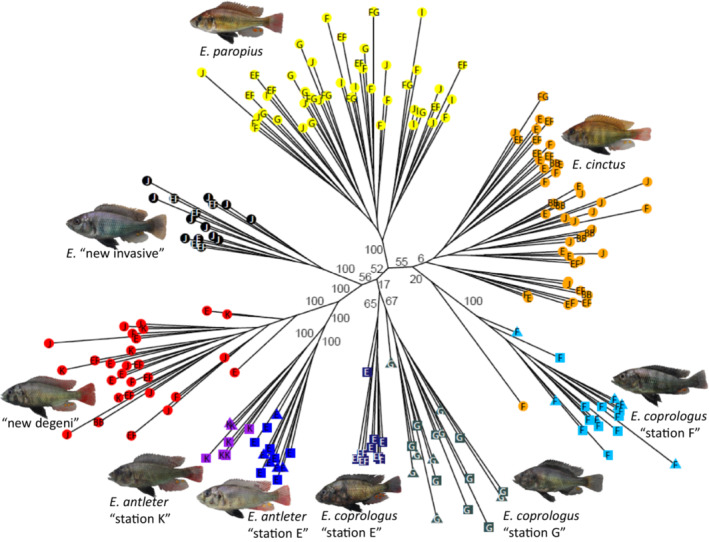
Phylogeny based on 28,376 biallelic SNPs allowing up to 90% of missing data. Tip colors represent species/population. Values at the branches represent bootstrap support from 100 rounds of bootstrapping using RAxML's rapid bootstrap algorithm. Bootstrap support values within species are not shown. Letters within branch tips refer to catch stations; squares indicate blue morphs; and triangles indicate red morphs.

None of the forms that we hypothesized to be male color morphs of the same species were genomically differentiated in the PCA on SNPs, in the STRUCTURE analysis, by significant *F*_ST_ values or in the RaxML tree (Figures 2, 3, 4, Figure S3), nor did they show differentiation in morphology by LDA (Table S3). Hence, in all subsequent analyses, we pooled the male color morphs within both species (one population per species and sampling site).

### 3.2 Species are distinct monophyletic entities in full sympatry

In the PCA based on the SNPs, PC1 and PC2 combined differentiated most of the sympatric putative species irrespective of sampling location (Figure 2). The molluscivore “new degeni” is separated by PC1 from the detritivorous species, all of which are clustered by putative species supporting phenotypic species diagnoses (see study species in Material & Methods). Surprisingly, the putative species *E. coprologus* was split into three clusters, mostly by sampling station. In the following, we referred to these as *E. coprologus* “station E” with individuals from station E and from a trawl shot done between station E and station F, *E. coprologus* “station F” and *E. coprologus* “station G”. Especially *E. coprologus* “station F” is differentiated from all other species on both genomic PC1 and PC2 and is clearly a very different species. *Enterochromis coprologus* from stations G and E were more similar to each other and each other's sister taxa in the phylogeny. We hence consider them populations of the same species, *E. coprologus*. Higher PCs did not reveal any additional cluster separations (Figure S4).

The STRUCTURE analysis with the Evanno Δ*K* method (which indicates the most likely *K* based on the largest change in magnitude of the second‐order rate of change in ln Pr(X|K) against successive *K* values) gave an optimum of *K* = 8, supporting the eight clusters found by the PCA (Figure 3). All species shared alleles with each other to some extent indicating moderate levels of admixture or incomplete lineage sorting. Remarkably, most of the shared alleles in *E. antleter* were with the molluscivore “new degeni”, which might be an indication of introgressive hybridization. Furthermore, *E. coprologus* “station F” is assigned to the most distinctly separate cluster from all other individuals with the least mixing. The two *E. cinctus* individuals and the *E. coprologus* “station G” individual showing the highest number of shared alleles in the STRUCTURE plot (Figure 3) also deviated the most from their respective species/population cluster in the PCA plot (Figure 2) and could potentially be F1 hybrids between *E. cinctus* and *E. coprologus*.

All the pairwise *F*_ST_ values between putative species revealed significant genomic differentiation between species. Differentiation levels between sympatric species ranged from 0.022 between *E. coprologus* “station G” and *E. paropius* to 0.099 between *E. coprologus* “station F” and “new degeni” (Figure S5, Table S4). The highest *F*_ST_ values showed that the highest levels of differentiation are found between *E. coprologus* “station F” and “new degeni”, supporting the result of the PCA.

The phylogenetic analysis showed clear species‐specific clades and most species were reciprocally monophyletic with bootstrap support of 100% (Figure 4). The *E. cinctus* branch had a low bootstrap support of 6%. Low bootstrap support was also found for the monophyly of *E. coprologus* “Station E” plus *E. coprologus* “Station G” which, together with significant allele sharing as seen in Figure 3, supports the possibility of hybridization between these putative species.

**FIGURE 5:**
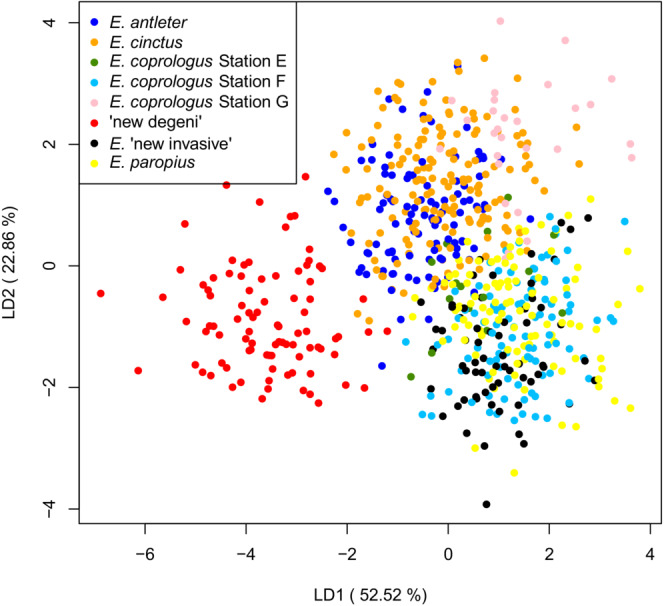
LDA plot showing the morphological differentiation along LD1 and LD2 between individuals based on 14 morphological characters with the percentage of variance explained in parentheses. Different symbols represent individuals from different species as indicated in the legend.

The LDA on morphology revealed a clustering of nine groups with the molluscivore “new degeni” being the morphologically most distinct species separated by LD1 (explaining 52.5% of the morphological variation, Figure 5, Table S5). Although the detritivorous species grouped together in the LDA plot and are not separated by LD 1, LD 2 (22.8%) separated *E. paropius*, *E*. “new invasive”, *E. coprologus* “station E”, and *E. coprologus* “station F” from *E. antleter*, *E. cinctus*, and *E. coprologus* “station G”. Almost all putative species differed significantly from each other in the LDA (LD1 and LD2). No significant differences were found between *E. antleter* “station E” and *E. cinctus* and between *E. paropius* and *E. coprologus* “station F.” These four putative species were, however, morphologically separated from each other by LD3 (10.5%, *p* < .001, Figure S6; Table S6).

### 3.3 Genetic and morphological differentiation within species between stations

Examining intraspecific genetic structure between sampling stations based on the genomic PCA revealed differentiation between *E. antleter* of stations E and K (opposite sides of the Mwanza Gulf) by PC6 (Figure S7), besides the differentiation between *E. coprologus* from stations E, F, and G (see above). The STRUCTURE analysis confirmed that *E. antleter* from station E and station K showed some differentiation (Figure 3). *Enterochromis antleter* from station E shares relatively more alleles with *E. cinctus*, while *E. antleter* from station K shares more alleles with *E. coprologus* “station E”. The *E. coprologus* from stations E and G share a considerable number of alleles with each other (dark blue and gray in Figure 3), consistent with the results of the PCA. It is noteworthy that *E. coprologus* “station G” shares many alleles with *E. paropius*, the most common detritivore at station G (and the entire deep water section of the transect, Figure 1).

**FIGURE 6:**
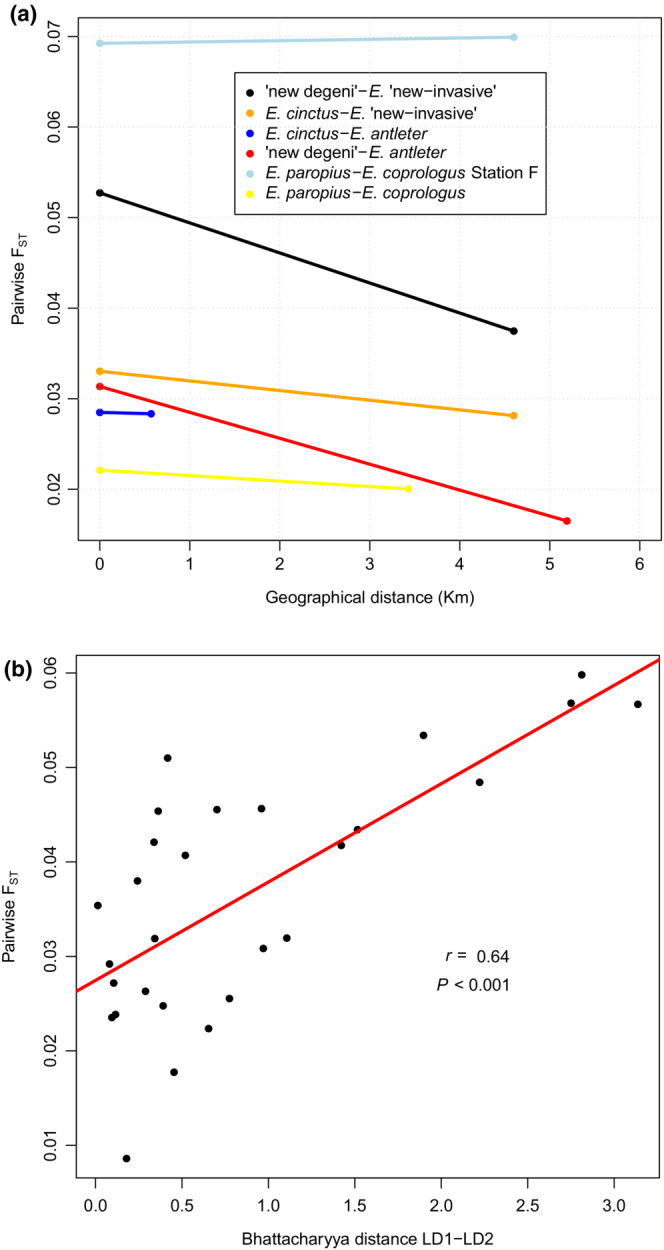
(a) Geographical distance plotted against pairwise *F*_ST_ values of species/population pairs that occur both in sympatry (=0 km) and allopatry (>0 km). *F*_ST_ values of species pairs in sympatry are always higher or similar compared to those in allopatry. (b) Bhattacharyya distance of LD1 and LD2 (Table S5) plotted against pairwise *F*_ST_ values of closely related species/population pairs (Table S4). Note that *E. coprologus* “station F” is not included in this graph.

Pairwise *F*_ST_ values between conspecific populations from different trawling stations did not reveal any significant differentiation except in *E. coprologus* (station G vs. station E; *F*_ST_ = 0.018, *p* < .001; Figure S8) and *E. antleter* (station E vs. station K; *F*_ST_ = 0.008, *p* < .001; Figure S8). In both cases, we saw morphological differentiation between the populations from these sampling stations too (*p* < .001; Table S5). Isolation by distance (IBD) was not significant for “new degeni” (*p* = .81) but was for *E. paropius* (*p* = .04) and *E. cinctus* (*p* = .009). We could not test for IBD within *E. coprologus*, *E. antleter*, or *E. “*new invasive” as these species/populations were not sampled at three or more different stations. We compared pairwise *F*_ST_ values of species that both occur in syntopy (at the same sampling station) and para‐ or allopatry (at different sampling stations). This showed that *F*_ST_ values among sympatric populations of widely distributed species were similar or higher than between the same two species in allopatry (Figure 6a).

The phylogenetic analysis revealed bootstrap support of 100% for *E. antleter* from station E and station K being distinct populations, while it was much lower for *E. coprologus* from stations E and G, 65% and 67%, respectively (Figure 4). In none of the other species could we detect any indications of geographical genetic structure. The phylogenetic analysis corroborates the results of the PCA, STRUCTURE, and the pairwise *F*_ST_ in providing support for species delimitation and genetic stability of species across multiple sites/sampling station.

### 3.4 Genetic vs. morphological differentiation

The subtle morphological differentiation and the clear genetic differentiation between species were plotted against each other to visualize the correlation between both. We decided to exclude *E. coprologus* “station F” from this analysis because it seemed more distantly related to the other species in this study (see Table S4, Figure 2). A significant positive correlation (*r* = 0.64, *p* < .001) between morphological and genetic differentiation was found (Figure 6b). Note that this correlation is less strong when *E. coprologus* “station F” was included in this analysis.

## 4 DISCUSSION

Reciprocal monophyly among species typically emerges only at late stages of speciation (Nosil et al., 2009). Lake Victoria cichlid fish are the most rapidly radiating group of animals known. The ~500 endemic species must have arisen in the past 16,000 years (Johnson et al., 1996; Meier, Sousa, et al., 2017). Three papers had previously investigated reciprocal monophyly of closely related species and all three found it strongly supported (Keller et al., 2013; Meier, Marques, et al., 2017; Wagner et al., 2013). All of these papers investigated reciprocal monophyly of species within individual communities. None of them addressed whether species' reciprocal monophyly is stable across species' geographical ranges that generally extend well beyond a single location and community. Because of interspecific hybridization and the potential parallel evolution of similar forms in different locations, this need not be the case (Feder et al., 2012). Here, we begin to test for Lake Victoria cichlid species monophyly across a wider geographical range with changing community composition. We studied the offshore assemblage of demersal (bottom‐dwelling) cichlids of the northern Mwanza Gulf. With the exception of *Enterochromis coprologus*, which we discovered to include two species (both monophyletic and not closely related), we found all phenotypically diagnosed species to be reciprocally monophyletic across their sampled geographical ranges. Besides our phylogenetic analysis, we observe the genomic differentiation among species also in the PCA, STRUCTURE analysis, and *F*_ST_ values. All population genomic analyses corroborate the phenotypic diagnoses and genomic stability of species across their range. Since all these species occur in sympatry in the broad sense and most of them occur in syntopy (same water depth at the same sampling station), the strong genetic clustering and reciprocal monophyly imply that all of these forms that differ distinctively in male nuptial coloration and subtly in morphology (plus one pair of distantly related species that differ in morphology but not or only subtly in nuptial coloration) are distinct species. On the other hand, none of our analyses detected differentiation between the red and blue male color morphs that we observed within populations of *E. antleter* and *E. coprologus* and that did not differ in morphology.

Geographical differentiation between conspecific populations was mostly weak or absent (Figures 3 and 4), and generally much weaker than the non‐geographical differentiation between species. *Enterochromis antleter* and *E. coprologus* “G/E” were the only two species that showed significant genomic differentiation between populations from different sampling stations. For *E. antleter* from stations E and K, the genomic differentiation is most likely the result of isolation caused by ~5 km of unfavorable habitat (deep water) separating the littoral on the two sides of the Mwanza Gulf. We, therefore, consider these as distinct allopatric populations of one species. The genomic differentiation between *E. coprologus* from stations E and G could also be caused by geographical isolation. However, since both genomic and morphological differentiation are more pronounced compared to that of the allopatric populations of *E. antleter*, the distance between the two stations is considerably less (~2 km), and intervening habitat does not seem unsuitable; it seems possible that what we consider *E. coprologus* from stations E and G are actually distinct sister species. More detailed morphological analyses and additional sampling to explore possible geographical overlap could perhaps help answer this question in the future.

Two species showed evidence of isolation by distance (IBD), *E. paropius* and *E. cinctus*. However, we cannot rule out that this signal alternatively results from geographically heterogeneous introgression. *Enterochromis* sp. “new invasive” does not show any sign of geographical structure, although this species too was caught at opposite sides of the Mwanza Gulf transect (stations E‐F and J) and was absent at the deeper stations in between. *Enterochromis* sp. “new invasive” was only discovered by OS in 2003 outside the Mwanza Gulf, and was first observed on the research transect in 2005. Because extensive sampling had been carried out on this transect for several decades before (Witte et al., 1992), it is very unlikely that this species had been overlooked in earlier years. *Enterochromis* sp. “new invasive” may have invaded southeastern Lake Victoria from elsewhere during the 2000s when haplochromine numbers in the offshore habitats recovered after two decades of extremely low numbers (Witte et al., 2007). Alternatively, this species could be a recently emerged hybrid taxon that rapidly spread. Either of these hypotheses could explain the lack of detectable geographical population structure in this recently expanding taxon. Based on its morphology, *E*. “new invasive” could have arisen from hybridization between some *Enterochromis* and a zooplanktivore of the slender *Yssichromis* genus. Species of *Yssichromis* were the first to recover in the region in the early 1990s after the near extinction (Witte et al., 2000). This hypothesis will have to be tested taxonomically with more inclusive sampling in the future.

### 4.1 Hybridization

The young age of the Lake Victoria Species Flock (<15,000 years; Johnson et al., 1996) and its hybrid origins (Meier, Marques, et al., 2017) are likely to have resulted in much lineage sharing among species. At the same time, hybridization has increased in recent years in the increasingly turbid waters of the Mwanza Gulf (Konijnendijk et al., 2011), making delineating species based on phenotypes even more difficult now than it used to be before the near extinction. Among our 198 individuals, 3 show a very high extent of admixture between species which is likely the result of recent hybridization, with these individuals being early generation, perhaps F1 hybrids (red circles in Figure 2; arrows in Figure 3). Indications of recent but not necessarily ongoing hybridization are seen also in our data for the species “new degeni.” This taxon differs in female coloration, stripe pattern, and morphology from *Platytaeniodus degeni* from before the near extinction. Both the body shape and the coloration of “new degeni” resemble that of *E. antleter*. We observe a large fraction of shared ancestry between these two taxa, pointing to hybridization in the past (Figure 3).

### 4.2 Species diverge in morphology after speciation

The shape of the relationship between genomic and morphological distance between our species suggests that morphology diverges only after speciation happened and genome‐wide genetic differentiation has started to accumulate. Had divergent selection on morphology initiated the speciation process, we would have expected the morphological distance to increase faster than genetic distance early in the process. This is because selection will only affect those regions in the genome that contain variants coding for the phenotypes under selection. The remainder of the genome would only begin to accumulate allelic divergence as a consequence of the cessation of gene flow once the selection‐driven phenotypic divergence had already caused reproductive isolation. We do not see evidence for this in our data.

Lake Victoria haplochromines have strongly sexually dimorphic coloration with bright colorful male breeding dress and cryptically colored females (Seehausen, 1996). Male coloration in these fish is under sexual selection by female mate choice (Maan et al., 2004) and divergence in male signals and female preferences are known to be important in speciation of rocky shore cichlids (Seehausen, 2015; Seehausen et al., 2008; Selz et al., 2016). The sympatric species of demersal detritivores are ecologically and morphologically very similar but have very different male nuptial coloration. It is tempting to speculate that sexual selection plays a key role in speciation in these demersal detritivores too, possibly interacting with divergent selection on the visual system between depths habitats. Consistent with this, we recently found evidence in rocky shore cichlids of the genus *Pundamilia* that divergent selection on feeding‐related morphological traits becomes effective only after speciation has already happened (van Rijssel et al., 2018). As the *Pundamilia* species also differ mainly in male nuptial coloration (although ecological and morphological differences are more pronounced than in the demersal detritivores), it seems possible that a similar speciation mechanism might be applicable to the demersal detritivores.

## 5 CONCLUSION

Strong genetic differentiation of species in full sympatry and their reciprocal monophyly that is stable across the sampled geographical range of species confirm that the closely related and ecologically similar species within the assemblage of offshore demersal detritivorous cichlids in Lake Victoria are distinct species at an advanced stage of speciation rather than incipient species or locally adapted populations. The shape of the relationship between genomic and morphological divergence among these species is consistent with the interpretation that, rather than driving speciation, morphological divergence happens after speciation has already occurred.

## AUTHOR CONTRIBUTIONS

**Jacco Van Rijssel:** Conceptualization (equal); formal analysis (lead); methodology (lead); visualization (lead); writing – original draft (lead); writing – review and editing (lead). **Florian Moser:** Data curation (supporting); formal analysis (supporting); methodology (supporting); writing – original draft (supporting); writing – review and editing (supporting). **Salome Mwaiko:** Formal analysis (supporting); methodology (supporting). **Ole Seehausen:** Conceptualization (lead); funding acquisition (lead); supervision (lead); writing – original draft (equal); writing – review and editing (equal).

## CONFLICT OF INTEREST

We declare we have no competing interests.

## Supporting information


Appendix S1:

## Data Availability

Data on morphology will be made available on Dryad. The RAD‐seq data will be made available on GenBank.
