# Supplementary material for: Strong species structure but weak geographical structure in demersal Lake Victoria cichlids
Source: Ecol Evol. 2022 Dec 25;12(12):e9669. doi: 10.1002/ece3.9669 (PMC9790821; doi:10.1002/ece3.9669)
Supplement: Supplementary file 1 — Appendix S1: [file ECE3-12-e9669-s001.docx]

Table S1. Number of individuals per species and colour morph per station used for morphometric measurements.

| Species / station | BB (4m) | E (6m) | E-F (7m) | E-F (9m) | F (10m) | F-G (12m) | G (14m) | I (10m) | J (6m) | K (4m) | Total |
| --- | --- | --- | --- | --- | --- | --- | --- | --- | --- | --- | --- |
| *E. antleter* "blue" |  | 50 |  | 1 |  | 1 | 1 |  | 2 | 17 | 72 |
| *E. antleter* "red" |  | 35 | 4 |  | 1 |  |  |  |  | 5 | 45 |
| *E. cinctus* | 10 | 53 | 35 | 3 | 32 | 1 |  |  | 14 |  | 148 |
| *E. coprologus* station E "blue" |  | 8 | 5 | 1 |  |  |  |  |  |  | 14 |
| *E. coprologus* station E "red" |  | 2 | 3 | 3 |  |  |  |  |  |  | 8 |
| *E. coprologus* station F "blue" |  |  |  |  | 40 |  |  |  |  |  | 40 |
| *E. coprologus* station F "red" |  |  |  |  | 48 |  |  |  |  |  | 48 |
| *E. coprologus* station G "blue" |  |  |  |  |  |  | 17 |  |  |  | 17 |
| *E. coprologus* station G "red" |  |  |  |  |  |  | 14 |  |  |  | 14 |
| ”new degeni” | 1 | 10 | 11 | 12 | 16 |  |  |  | 29 | 10 | 89 |
| *E.* 'new-invasive' |  | 1 | 6 |  |  |  |  |  | 61 |  | 68 |
| *E. paropius* |  |  |  |  | 25 |  | 25 | 25 | 23 |  | 98 |
| Total | 11 | 159 | 64 | 20 | 162 | 2 | 54 | 27 | 129 | 33 | 661 |

Table S2. Number of individuals per species and colour morph per station used for RAD-sequencing.

| Species / station | BB (4m) | E (6m) | | E-F (7m) | E-F (9m) | F (10m) | F-G (12m) | G (14m) | I (10m) | J (6m) | K (4m) | Total |
| --- | --- | --- | --- | --- | --- | --- | --- | --- | --- | --- | --- | --- |
| *E. antleter* "blue" |  | 7 |  | |  |  |  |  |  |  | 5 | 12 |
| *E. antleter* "red" |  | 5 |  | |  |  |  |  |  |  | 2 | 7 |
| *E. cinctus* | 7 | 11 | 8 | |  | 8 |  |  |  | 12 |  | 46 |
| *E. coprologus* station E "blue" |  | 5 | 4 | |  |  |  |  |  |  |  | 9 |
| *E. coprologus* station F "blue" |  |  |  | |  | 10 |  |  |  |  |  | 10 |
| *E. coprologus* station F "red" |  |  |  | |  | 7 |  |  |  |  |  | 7 |
| *E. coprologus* station G "blue" |  |  |  | |  |  |  | 9 |  |  |  | 9 |
| *E. coprologus* station G "red" |  |  |  | |  |  |  | 8 |  |  |  | 8 |
| “new degeni” | 1 | 6 | 5 | |  | 3 |  |  |  | 8 | 5 | 28 |
| *E.* 'new-invasive' |  |  | 7 | |  |  |  |  |  | 9 |  | 16 |
| *E. paropius* |  |  |  | | 7 | 9 | 5 | 8 | 7 | 10 |  | 46 |
| Total | 8 | 34 | 24 | | 7 | 37 | 5 | 25 | 7 | 39 | 12 | 198 |

Table S3. Bhattacharyya distances (Bd) of LD1 and LD2 of the linear discriminant function analysis between nine species/populations including the colour morphs. Significant P-values after sequential Bonferroni correction are depicted in bold. None of the colour morphs were morphologically significantly different within species.

| Species/population/morph | Species/population/morph | bd | P-value |
| --- | --- | --- | --- |
| *E. antleter* blue "station E" | *E. antleter* blue "station K" | 0.222896 | **<0.001** |
| *E. antleter* blue "station E" | *E. antleter* red "station E" | 0.02179 | 0.407909 |
| *E. antleter* blue "station E" | *E. antleter* red "station K" | 0.339691 | 0.259495 |
| *E. antleter* blue "station E" | *E. cinctus* | 0.017375 | 0.458195 |
| *E. antleter* blue "station E" | *E. coprologus* blue "station E" | 0.470067 | **<0.001** |
| *E. antleter* blue "station E" | *E. coprologus* red "station E" | 0.300161 | **0.004178** |
| *E. antleter* blue "station E" | *E. coprologus* blue "station F" | 0.996215 | **<0.001** |
| *E. antleter* blue "station E" | *E. coprologus* red "station F" | 0.726167 | **<0.001** |
| *E. antleter* blue "station E" | *E. coprologus* blue "station G" | 0.716315 | **<0.001** |
| *E. antleter* blue "station E" | *E. coprologus* red "station G" | 0.471362 | **<0.001** |
| *E. antleter* blue "station E" | ”new degeni” | 1.615224 | **<0.001** |
| *E. antleter* blue "station E" | *E.* "new invasive" | 0.894371 | **<0.001** |
| *E. antleter* blue "station E" | *E. paropius* | 0.669178 | **<0.001** |
| *E. antleter* blue "station E" | *E. cinctus* | 0.501263 | **0.002337** |
| *E. antleter* blue "station K" | *E. antleter* red "station E" | 0.321244 | **<0.001** |
| *E. antleter* blue "station K" | *E. antleter* red "station K" | 0.570735 | 0.07849 |
| *E. antleter* blue "station K" | *E. coprologus* blue "station E" | 0.08897 | 0.10651 |
| *E. antleter* blue "station K" | *E. coprologus* red "station E" | 0.197544 | 0.714792 |
| *E. antleter* blue "station K" | *E. coprologus* blue "station F" | 0.42708 | **<0.001** |
| *E. antleter* blue "station K" | *E. coprologus* red "station F" | 0.255905 | **<0.001** |
| *E. antleter* blue "station K" | *E. coprologus* blue "station G" | 1.165243 | **<0.001** |
| *E. antleter* blue "station K" | *E. coprologus* red "station G" | 0.92271 | **<0.001** |
| *E. antleter* blue "station K" | ”new degeni” | 1.754228 | **<0.001** |
| *E. antleter* blue "station K" | *E.* "new invasive" | 0.314108 | **<0.001** |
| *E. antleter* blue "station K" | *E. paropius* | 0.265122 | **<0.001** |
| *E. antleter* red "station E" | *E. antleter* red "station K" | 0.36334 | 0.241972 |
| *E. antleter* red "station E" | *E. cinctus* | 0.020995 | 0.075495 |
| *E. antleter* red "station E" | *E. coprologus* blue "station E" | 0.651428 | **<0.001** |
| *E. antleter* red "station E" | *E. coprologus* red "station E" | 0.424389 | **<0.001** |
| *E. antleter* red "station E" | *E. coprologus* blue "station F" | 1.266413 | **<0.001** |
| *E. antleter* red "station E" | *E. coprologus* red "station F" | 0.948726 | **<0.001** |
| *E. antleter* red "station E" | *E. coprologus* blue "station G" | 0.835085 | **<0.001** |
| *E. antleter* red "station E" | *E. coprologus* red "station G" | 0.559361 | **<0.001** |
| *E. antleter* red "station E" | ”new degeni” | 1.417064 | **<0.001** |
| *E. antleter* red "station E" | *E.* "new invasive" | 1.181394 | **<0.001** |
| *E. antleter* red "station E" | *E. paropius* | 0.853083 | **<0.001** |
| *E. antleter* red "station K" | *E. cinctus* | 0.387152 | 0.049217 |
| *E. antleter* red "station K" | *E. coprologus* blue "station E" | 1.095046 | **0.002598** |
| *E. antleter* red "station K" | *E. coprologus* red "station E" | 0.888792 | 0.103176 |
| *E. antleter* red "station K" | *E. coprologus* blue "station F" | 2.40488 | **<0.001** |
| *E. antleter* red "station K" | *E. coprologus* red "station F" | 1.566986 | **<0.001** |
| *E. antleter* red "station K" | *E. coprologus* blue "station G" | 1.200507 | **<0.001** |
| *E. antleter* red "station K" | *E. coprologus* red "station G" | 0.965063 | **0.001556** |
| *E. antleter* red "station K" | ”new degeni” | 1.207956 | **<0.001** |
| *E. antleter* red "station K" | *E.* "new invasive" | 1.856787 | **<0.001** |
| *E. antleter* red "station K" | *E. paropius* | 1.417166 | **<0.001** |
| *E. cinctus* | *E. coprologus* blue "station E" | 0.571044 | **<0.001** |
| *E. cinctus* | *E. coprologus* red "station E" | 0.372612 | **<0.001** |
| *E. cinctus* | *E. coprologus* blue "station F" | 1.097891 | **<0.001** |
| *E. cinctus* | *E. coprologus* red "station F" | 0.849166 | **<0.001** |
| *E. cinctus* | *E. coprologus* blue "station G" | 0.619442 | **<0.001** |
| *E. cinctus* | *E. coprologus* red "station G" | 0.392942 | **<0.001** |
| *E. cinctus* | ”new degeni” | 1.844151 | **<0.001** |
| *E. cinctus* | *E.* "new invasive" | 1.107774 | **<0.001** |
| *E. cinctus* | *E. paropius* | 0.7673 | **<0.001** |
| *E. coprologus* blue "station E" | *E. coprologus* red "station E" | 0.226663 | 0.738421 |
| *E. coprologus* blue "station E" | *E. coprologus* blue "station F" | 0.137434 | **0.00778** |
| *E. coprologus* blue "station E" | *E. coprologus* red "station F" | 0.070365 | 0.102444 |
| *E. coprologus* blue "station E" | *E. coprologus* blue "station G" | 0.925677 | **<0.001** |
| *E. coprologus* blue "station E" | *E. coprologus* red "station G" | 0.806381 | **<0.001** |
| *E. coprologus* blue "station E" | ”new degeni” | 2.248896 | **<0.001** |
| *E. coprologus* blue "station E" | *E.* "new invasive" | 0.126219 | 0.010508 |
| *E. coprologus* blue "station E" | *E. paropius* | 0.105714 | 0.071813 |
| *E. coprologus* red "station E" | *E. coprologus* blue "station F" | 0.361904 | **0.004359** |
| *E. coprologus* red "station E" | *E. coprologus* red "station F" | 0.234138 | 0.066354 |
| *E. coprologus* red "station E" | *E. coprologus* blue "station G" | 1.285453 | **<0.001** |
| *E. coprologus* red "station E" | *E. coprologus* red "station G" | 0.952813 | **<0.001** |
| *E. coprologus* red "station E" | ”new degeni” | 2.897036 | **<0.001** |
| *E. coprologus* red "station E" | *E.* "new invasive" | 0.206896 | 0.02415 |
| *E. coprologus* red "station E" | *E. paropius* | 0.239126 | 0.042498 |
| *E. coprologus* blue "station F" | *E. coprologus* red "station F" | 0.026256 | 0.389861 |
| *E. coprologus* blue "station F" | *E. coprologus* blue "station G" | 1.047767 | **<0.001** |
| *E. coprologus* blue "station F" | *E. coprologus* red "station G" | 0.998305 | **<0.001** |
| *E. coprologus* blue "station F" | ”new degeni” | 3.611965 | **<0.001** |
| *E. coprologus* blue "station F" | *E.* "new invasive" | 0.080224 | **0.001055** |
| *E. coprologus* blue "station F" | *E. paropius* | 0.047975 | 0.870025 |
| *E. coprologus* red "station F" | *E. coprologus* blue "station G" | 1.124328 | **<0.001** |
| *E. coprologus* red "station F" | *E. coprologus* red "station G" | 1.019595 | **<0.001** |
| *E. coprologus* red "station F" | ”new degeni” | 2.764845 | **<0.001** |
| *E. coprologus* red "station F" | *E.* "new invasive" | 0.038217 | 0.040354 |
| *E. coprologus* red "station F" | *E. paropius* | 0.015146 | 0.600893 |
| *E. coprologus* blue "station G" | *E. coprologus* red "station G" | 0.022459 | 0.687516 |
| *E. coprologus* blue "station G" | ”new degeni” | 4.771397 | **<0.001** |
| *E. coprologus* blue "station G" | *E.* "new invasive" | 1.655701 | **<0.001** |
| *E. coprologus* blue "station G" | *E. paropius* | 1.133376 | **<0.001** |
| *E. coprologus* red "station G" | ”new degeni” | 3.84269 | **<0.001** |
| *E. coprologus* red "station G" | *E.* "new invasive" | 1.50931 | **<0.001** |
| *E. coprologus* red "station G" | *E. paropius* | 1.004409 | **<0.001** |
| ”new degeni” | *E.* "new invasive" | 2.845072 | **<0.001** |
| ”new degeni” | *E. paropius* | 2.764768 | **<0.001** |
| *E.* "new invasive" | *E. paropius* | 0.079824 | **<0.001** |

Table S4. Pairwise genomic divergence (F_ST_) between all eight studied species/populations. The F_ST_ values are based on a dataset of 8 609 biallelic SNPs with at least 1 minor allele counts. The highest divergence is found between *Lithochromis* sp. and all species, while lowest diverence is between the the species/populations of *E. coprologus* from station E and G*.* All F_ST_ values between species/populations are significant (P < 0.05). Species abbrevations; ant = *E. antleter,* cin = *E. cinctus*, coE = *E. coprologus* “Station E”, coG = *E. coprologus* “Station G”, deg = ”new degeni”, coF = *E. coprologus* “Station F”, new = *E.* “new invasive”, par = *E. paropius*.

| **Species** | **ant** | **cin** | **coE** | **coF** | **coG** | **deg** | **new** | **par** |
| --- | --- | --- | --- | --- | --- | --- | --- | --- |
| **ant** | 0 |  |  |  |  |  |  |  |
| **cin** | 0.03456 | 0 |  |  |  |  |  |  |
| **coE** | 0.02735 | 0.02477 | 0 |  |  |  |  |  |
| **coF** | 0.08174 | 0.05335 | 0.06969 | 0 |  |  |  |  |
| **coG** | 0.03819 | 0.02631 | 0.01774 | 0.07571 | 0 |  |  |  |
| **deg** | 0.03921 | 0.0534 | 0.04843 | 0.09873 | 0.05669 | 0 |  |  |
| **new** | 0.04416 | 0.03194 | 0.02718 | 0.07674 | 0.03084 | 0.05981 | 0 |  |
| **par** | 0.04278 | 0.02554 | 0.02353 | 0.06503 | 0.02236 | 0.05681 | 0.0292 | 0 |

Table S5. Bhattacharyya distances (Bd) of LD1 and LD2 of the linear discriminant function analysis between nine species/populations. Significant P-values after sequential Bonferroni correction are depicted in bold.

| Species/population | Species/population | bd | P-value |
| --- | --- | --- | --- |
| *E. antleter* "Station E" | *E. antleter* "station K" | 0.17931 | **<0.001** |
| *E. antleter* "Station E" | *E. cinctus* | 0.013356 | 0.175564 |
| *E. antleter* "Station E" | *E. coprologus* "Station E" | 0.342917 | **<0.001** |
| *E. antleter* "Station E" | *E. coprologus* "Station F" | 0.857746 | **<0.001** |
| *E. antleter* "Station E" | *E. coprologus* "Station G" | 0.338217 | **<0.001** |
| *E. antleter* "Station E" | ”new degeni” | 1.51573 | **<0.001** |
| *E. antleter* "Station E" | *E.* "new invasive" | 0.960087 | **<0.001** |
| *E. antleter* "Station E" | *E. paropius* | 0.702321 | **<0.001** |
| *E. antleter* "station K" | *E. cinctus* | 0.242586 | **<0.001** |
| *E. antleter* "station K" | *E. coprologus* "Station E" | 0.115805 | **0.024421** |
| *E. antleter* "station K" | *E. coprologus* "Station F" | 0.434928 | **<0.001** |
| *E. antleter* "station K" | *E. coprologus* "Station G" | 0.519305 | **<0.001** |
| *E. antleter* "station K" | ”new degeni” | 1.421566 | **<0.001** |
| *E. antleter* "station K" | *E.* "new invasive" | 0.417149 | **<0.001** |
| *E. antleter* "station K" | *E. paropius* | 0.362517 | **<0.001** |
| *E. cinctus* | *E. coprologus* "Station E" | 0.390976 | **<0.001** |
| *E. cinctus* | *E. coprologus* "Station F" | 0.937916 | **<0.001** |
| *E. cinctus* | *E. coprologus* "Station G" | 0.288926 | **<0.001** |
| *E. cinctus* | ”new degeni” | 1.897069 | **<0.001** |
| *E. cinctus* | *E.* "new invasive" | 1.106339 | **<0.001** |
| *E. cinctus* | *E. paropius* | 0.774548 | **<0.001** |
| *E. coprologus* "Station E" | *E. coprologus* "Station F" | 0.108247 | **0.001053** |
| *E. coprologus* "Station E" | *E. coprologus* "Station G" | 0.454067 | **<0.001** |
| *E. coprologus* "Station E" | ”new degeni” | 2.221829 | **<0.001** |
| *E. coprologus* "Station E" | *E.* "new invasive" | 0.105239 | **0.002751** |
| *E. coprologus* "Station E" | *E. paropius* | 0.094466 | **0.005287** |
| *E. coprologus* "Station F" | *E. coprologus* "Station G" | 0.651345 | **<0.001** |
| *E. coprologus* "Station F" | ”new degeni” | 3.110278 | **<0.001** |
| *E. coprologus* "Station F" | *E.* "new invasive" | 0.055794 | **<0.001** |
| *E. coprologus* "Station F" | *E. paropius* | 0.021099 | 0.945762 |
| *E. coprologus* "Station G" | ”new degeni” | 3.136956 | **<0.001** |
| *E. coprologus* "Station G" | *E.* "new invasive" | 0.970524 | **<0.001** |
| *E. coprologus* "Station G" | *E. paropius* | 0.653934 | **<0.001** |
| ”new degeni” | *E.* "new invasive" | 2.812361 | **<0.001** |
| ”new degeni” | *E. paropius* | 2.750634 | **<0.001** |
| *E.* "new invasive" | *E. paropius* | 0.080738 | **<0.001** |

Table S6. Bhattacharyya distances (Bd) of LD3 and LD4 of the linear discriminant function analysis between nine species/populations. Significant P-values after sequential Bonferroni correction are depicted in bold.

| Species/population | Species/population | bd | P-value |
| --- | --- | --- | --- |
| *E. antleter* "Station E" | *E. antleter* "station K" | 0.179338 | **<0.001** |
| *E. antleter* "Station E" | *E. cinctus* | 0.076839 | **<0.001** |
| *E. antleter* "Station E" | *E. coprologus* "Station E" | 0.164607 | **<0.001** |
| *E. antleter* "Station E" | *E. coprologus* "Station F" | 0.397827 | **<0.001** |
| *E. antleter* "Station E" | *E. coprologus* "Station G" | 1.107198 | **<0.001** |
| *E. antleter* "Station E" | ”new degeni” | 0.139021 | **<0.001** |
| *E. antleter* "Station E" | *E.* "new invasive" | 0.13645 | **<0.001** |
| *E. antleter* "Station E" | *E. paropius* | 0.109344 | **<0.001** |
| *E. antleter* "station K" | *E. cinctus* | 0.382811 | **<0.001** |
| *E. antleter* "station K" | *E. coprologus* "Station E" | 0.061257 | 0.775327 |
| *E. antleter* "station K" | *E. coprologus* "Station F" | 0.672453 | **<0.001** |
| *E. antleter* "station K" | *E. coprologus* "Station G" | 0.560654 | **<0.001** |
| *E. antleter* "station K" | ”new degeni” | 0.187416 | **<0.001** |
| *E. antleter* "station K" | *E.* "new invasive" | 0.188953 | **<0.001** |
| *E. antleter* "station K" | *E. paropius* | 0.131593 | **<0.001** |
| *E. cinctus* | *E. coprologus* "Station E" | 0.249583 | **<0.001** |
| *E. cinctus* | *E. coprologus* "Station F" | 0.151532 | **<0.001** |
| *E. cinctus* | *E. coprologus* "Station G" | 1.196413 | **<0.001** |
| *E. cinctus* | ”new degeni” | 0.099109 | **<0.001** |
| *E. cinctus* | *E.* "new invasive" | 0.39461 | **<0.001** |
| *E. cinctus* | *E. paropius* | 0.099675 | **<0.001** |
| *E. coprologus* "Station E" | *E. coprologus* "Station F" | 0.388966 | **<0.001** |
| *E. coprologus* "Station E" | *E. coprologus* "Station G" | 0.567597 | **<0.001** |
| *E. coprologus* "Station E" | ”new degeni” | 0.155373 | **<0.001** |
| *E. coprologus* "Station E" | *E.* "new invasive" | 0.340521 | **<0.001** |
| *E. coprologus* "Station E" | *E. paropius* | 0.102539 | **0.008597** |
| *E. coprologus* "Station F" | *E. coprologus* "Station G" | 1.045529 | **<0.001** |
| *E. coprologus* "Station F" | ”new degeni” | 0.225637 | **<0.001** |
| *E. coprologus* "Station F" | *E.* "new invasive" | 1.00873 | **<0.001** |
| *E. coprologus* "Station F" | *E. paropius* | 0.239328 | **<0.001** |
| *E. coprologus* "Station G" | ”new degeni” | 0.627443 | **<0.001** |
| *E. coprologus* "Station G" | *E.* "new invasive" | 1.405612 | **<0.001** |
| *E. coprologus* "Station G" | *E. paropius* | 0.609421 | **<0.001** |
| ”new degeni” | *E.* "new invasive" | 0.356346 | **<0.001** |
| ”new degeni” | *E. paropius* | 0.007004 | 0.520473 |
| *E.* "new invasive" | *E. paropius* | 0.306709 | **<0.001** |


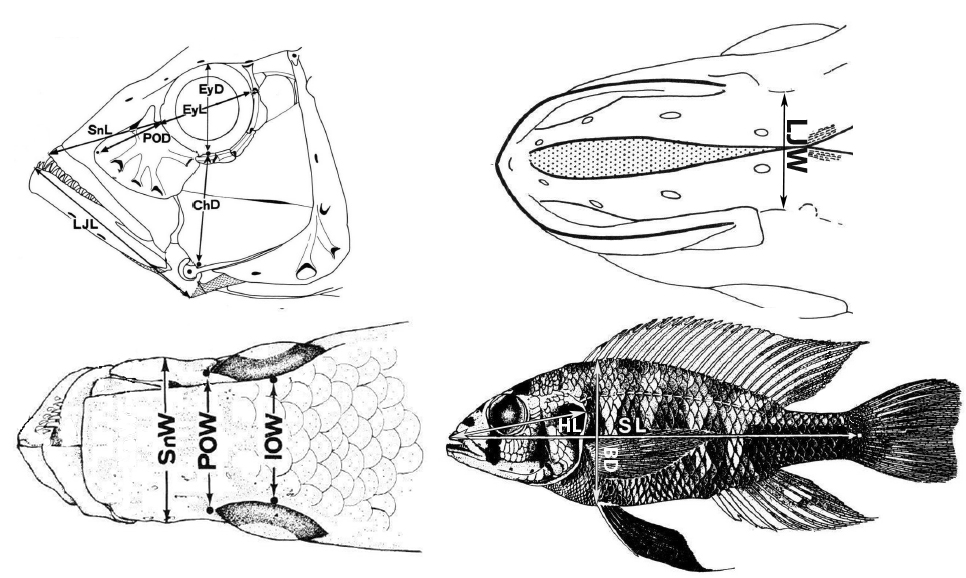


**PPL**

Figure S1. Morphometric measurements used in this study, modified from ([Barel *et al.* 1977](#_ENREF_1)). Note that head width (HW) is not indicated in this figure which is the width between the most caudal point of the left and right operculum, also used to measure head length (HL).

Barel CDN, van Oijen MJP, Witte F, Witte-Maas ELM (1977) Introduction to taxonomy and morphology of haplochromine cichlidae from Lake Victoria - Manual to Greenwoods revision papers. *Netherlands Journal of Zoology* **27**, 333-389.

Figure S2 Number of loci with a minimum read depth of 10 plotted against the number of mapped reads for all five libraries sequenced, before filtering steps.


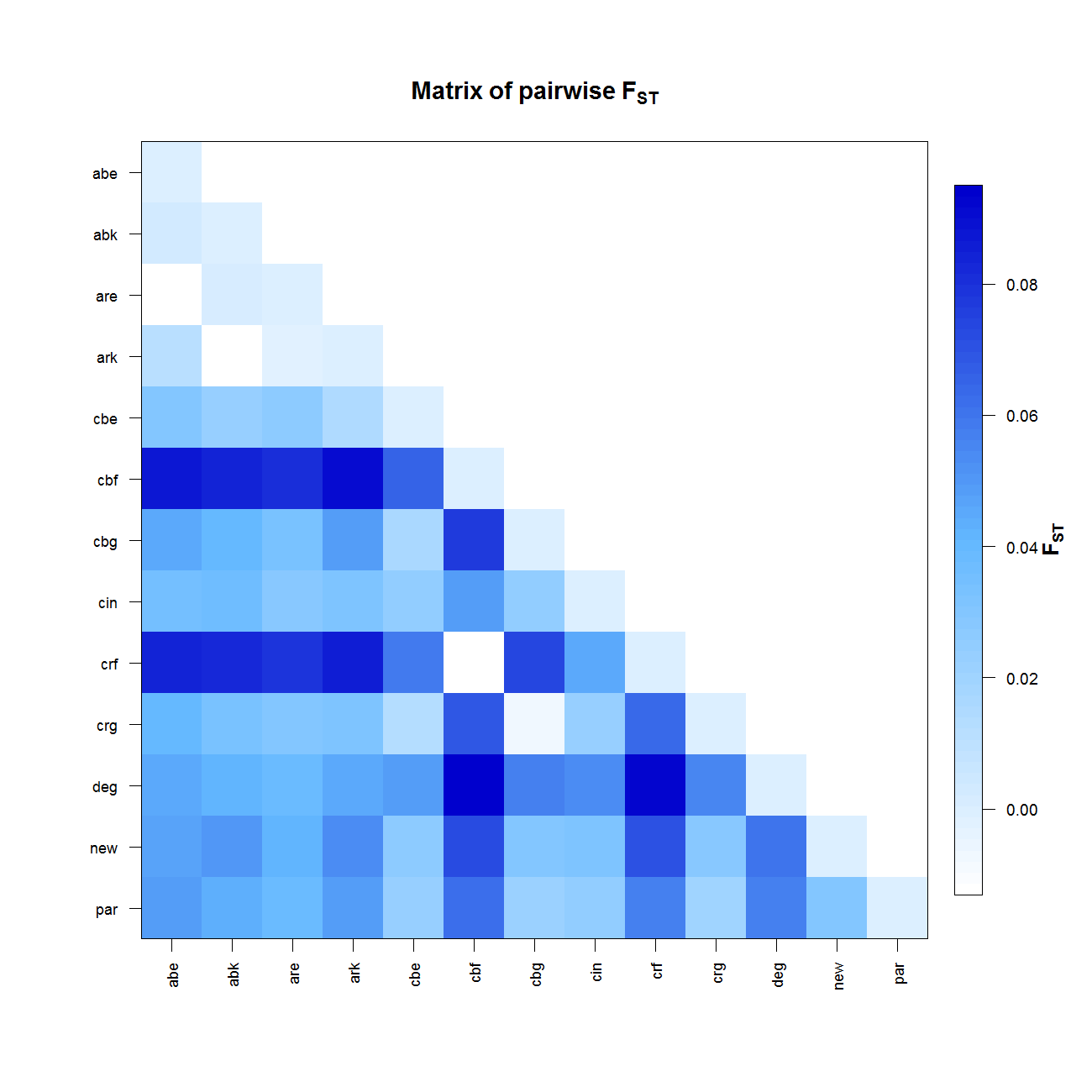


Figure S3. Pairwise divergence (F_ST_) between all eight studied species/populations and the red/blue colour morphs of *E. antleter*, *E. coprologus* “Statin “G” and *Lithochromis* sp. The F_ST_ values are based on a dataset of 8 609 biallelic SNPs with at least 1 minor allele count*.* None of F_ST_ values between colour morphs was significant. Note that we only sampled blue morphs of *E. coprologus* “Statin “E”. Species and colour morph abbrevations; abe = *E. antleter* blue “Station E”*,* abk = *E. antleter* blue “Station K”*,* are = *E. antleter* red “Station E”*,* ark = *E. antleter* red “Station K”*,* cin = *E. cinctus*, cbe = *E. coprologus* blue “Station E”, cbg = *E. coprologus* blue “Station G”, crg = *E. coprologus* red “Station G”, deg = ”new degeni”, cbf = *E. coprologus* blue "Station F", crf = *E. coprologus* blue "Station F", new = *E.* “new invasive”, par = *E. paropius*.


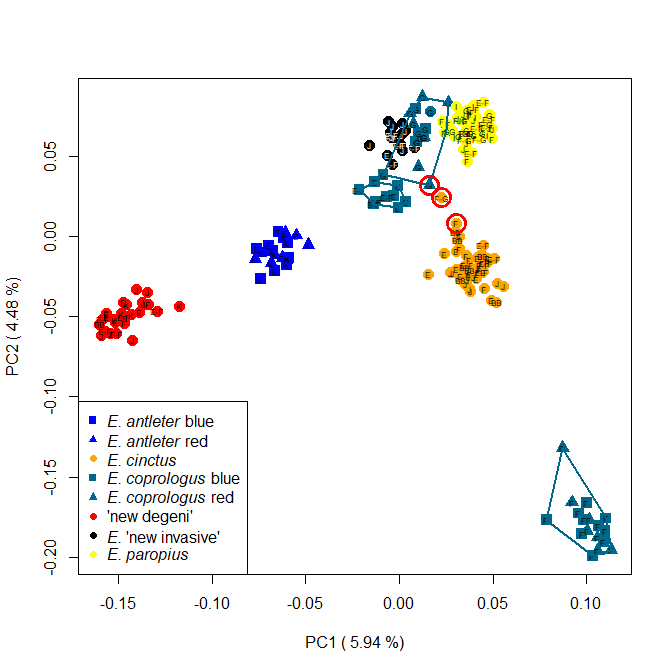

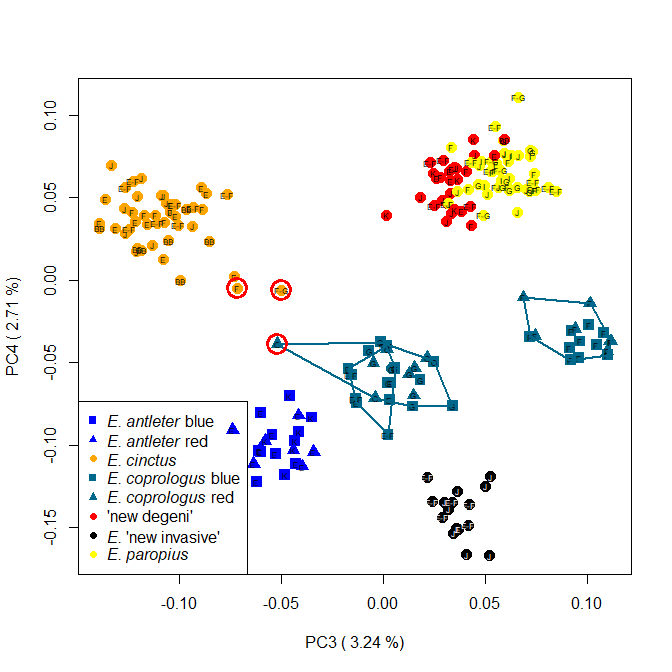


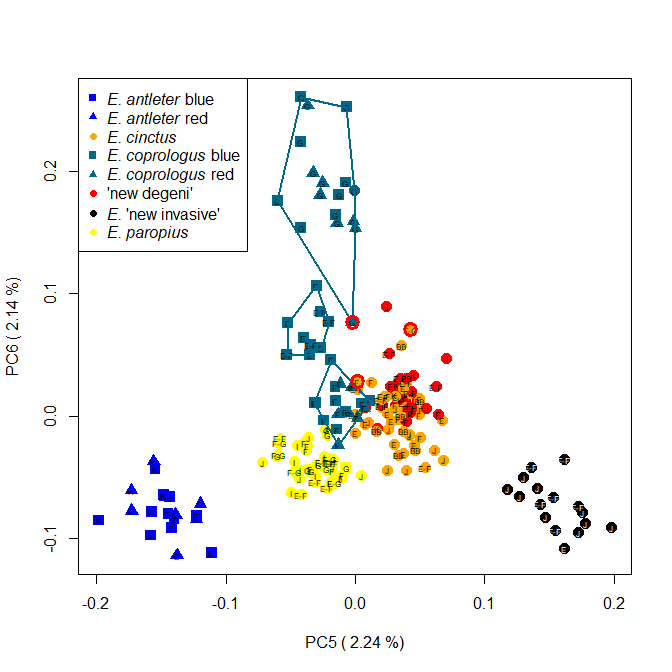

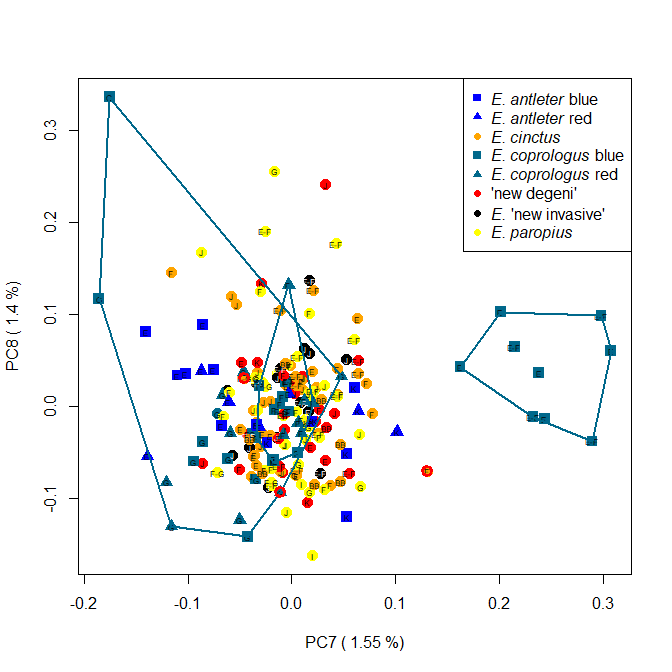


Figure S4. PCA plots showing the genetic differentiaton between sampled individuals based on 8 609 SNPs. The first eight axes are shown with the percentage of variance explained in parentheses. Different symbols represent individuals from different species and colour morphs as indicated in the legend. *Enterochromis coprologus* individuals from station E, F and G are indicated by convex hulls per station. Two individuals of *E. cinctus* and on individual from *E. coprologus* station G that share a relatively large amount of alleles are indicated with red circles. ”new degeni”


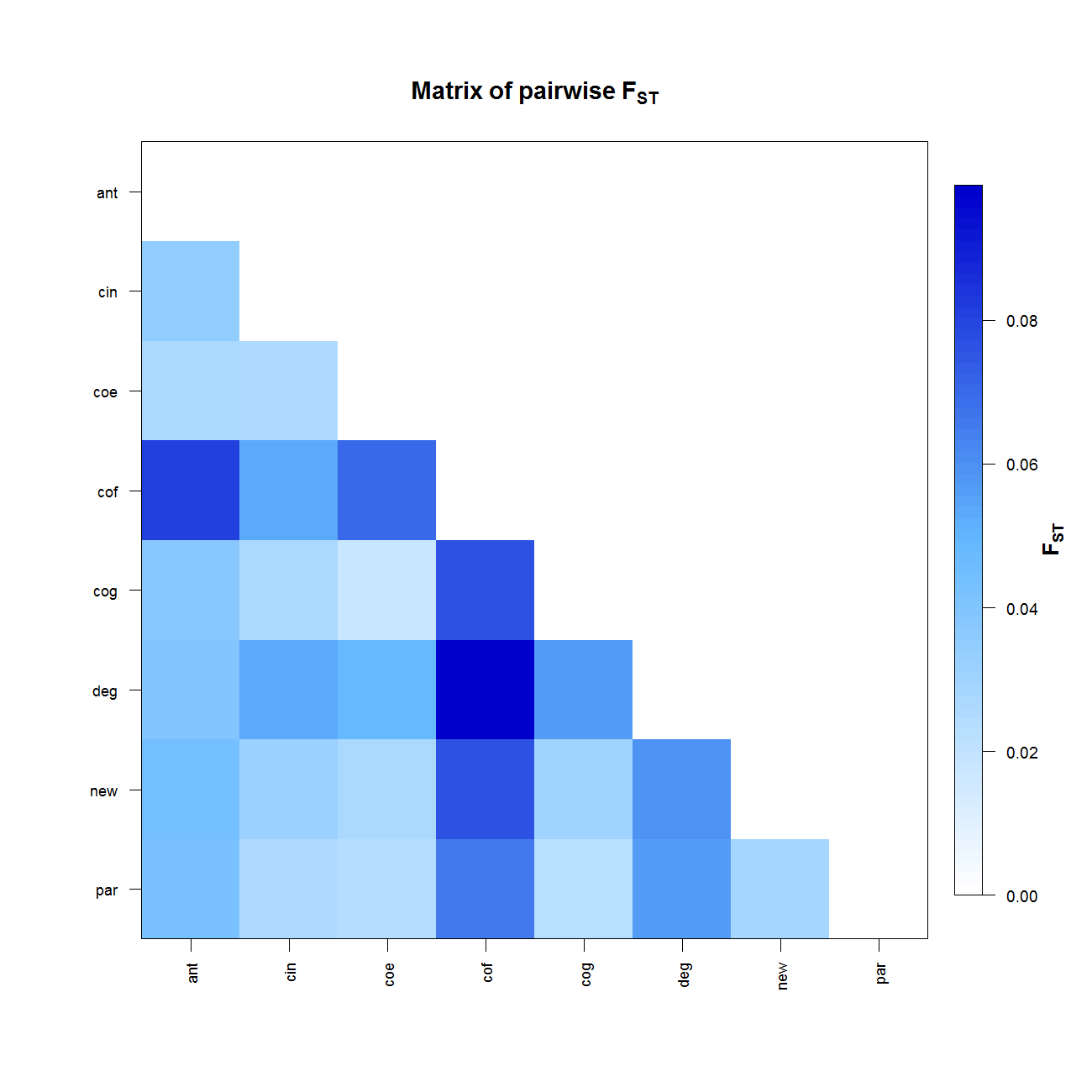


Figure S5. Pairwise genomic divergence (F_ST_) between all eight studied species/populations. The F_ST_ values are based on a dataset of 8 609 biallelic SNPs with at least 1 minor allele count. The highest divergence is found between *E. coprologus* "Station F" and all species, while lowest diverence is between the the species/populations of *E. coprologus* from station E and G*.* All F_ST_ values between species/populations are significant (P < 0.05). Species abbrevations; ant = *E. antleter,* cin = *E. cinctus*, coe = *E. coprologus* “Station E”, cog = *E. coprologus* “Station G”, deg = ”new degeni”, cof = *E. coprologus* "Station F", new = *E.* “new invasive”, par = *E. paropius*.


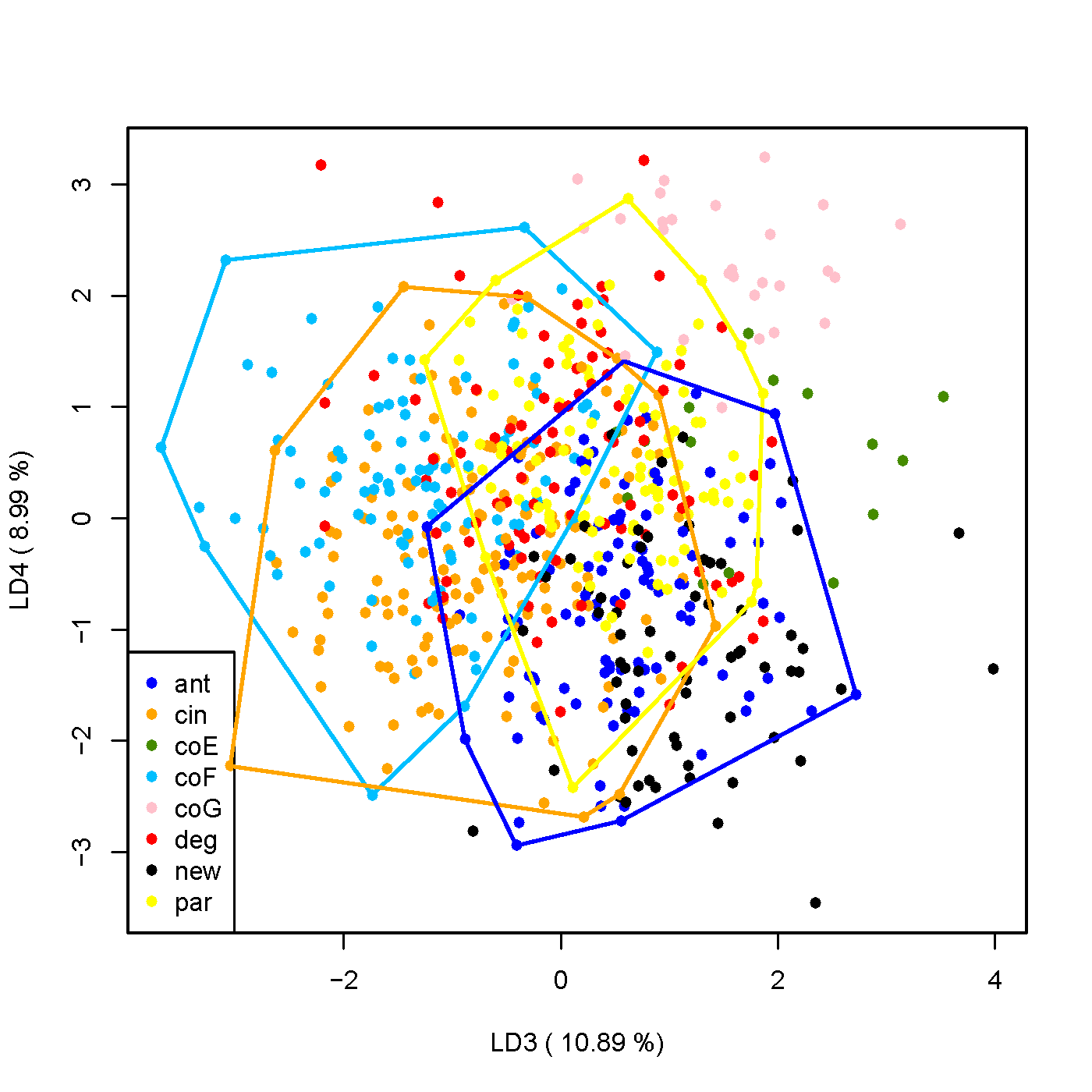


Figure S6. LDA plot showing the morphological differentiaton along LD3 and LD4 between measured individuals based on 14 morphological characters with the percentage of variance explained in parentheses. Different symbols represent individuals from different species and colour morphs as indicated in the legend; ant = *E. antleter,* cin = *E. cinctus*, coE = *E. coprologus* “Station E”, coG = *E. coprologus* “Station G”, deg = ”new degeni”, coF = *E. coprologus* "Station F", new = *E.* “new invasive”, par = *E. paropius*. LD3 separates species/populations of *E. antleter* “station E” and *E. cinctus* and the clusters of *E. paropius* and *E. coprologus* "Station F".. Convex hulls are added for *E. coprologus* "Station F", *E. cinctus*, *E. antleter* and *E. paropius* to illustrate subtle morphological differences on LD3.


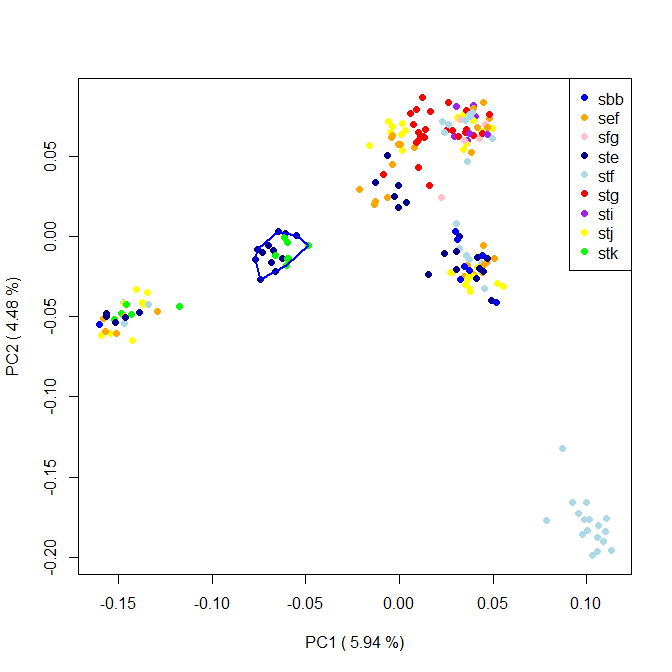

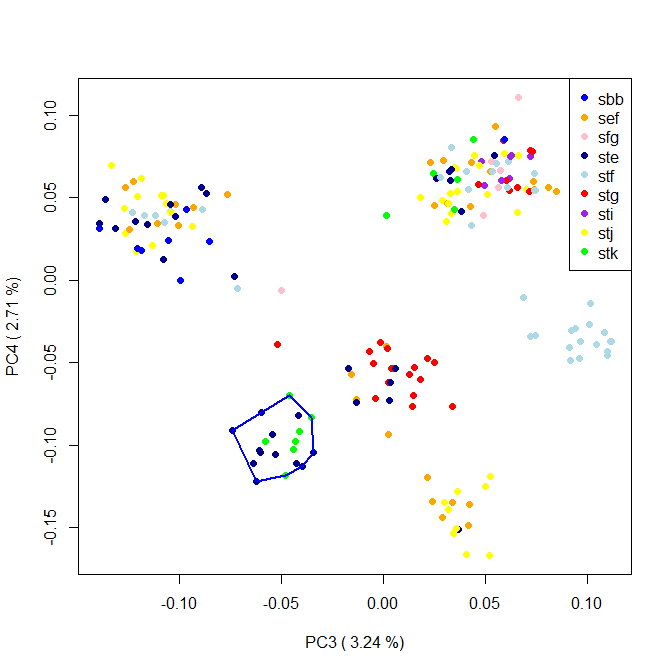

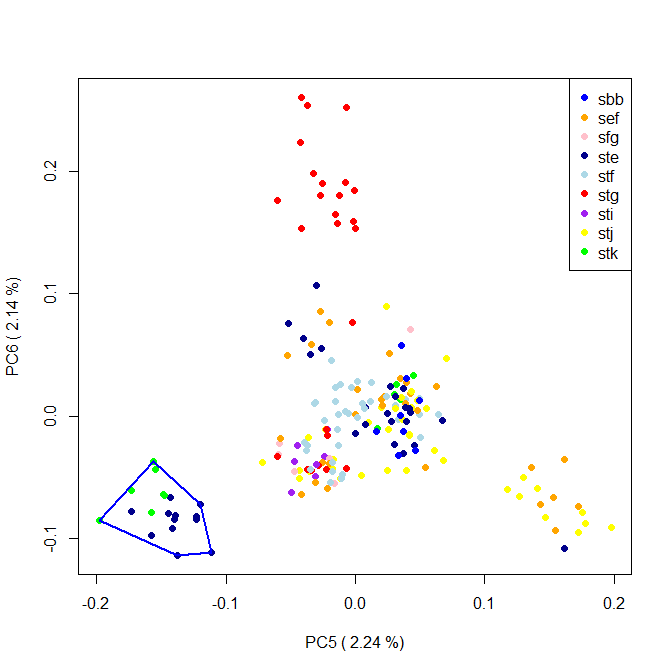

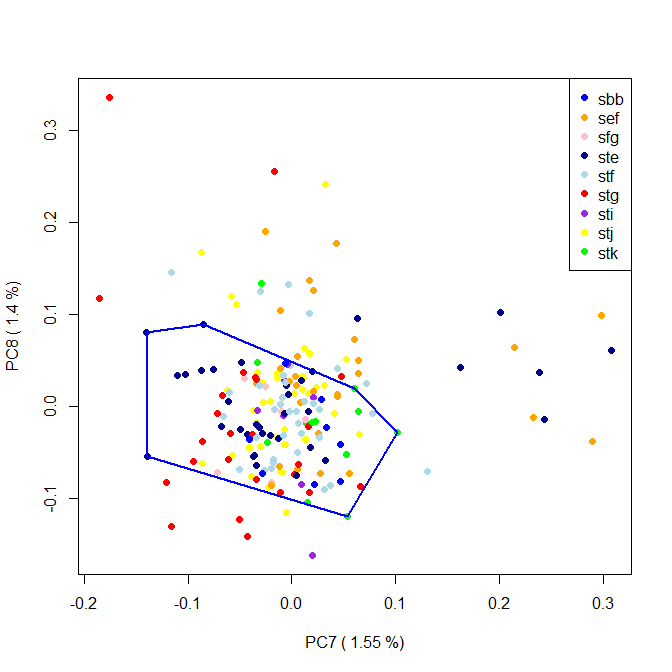


Figure S7. PCA plots showing the genetic differentiaton between sampled individuals coloured by station based on 8 609 biallelic SNPs. The first eight axes are shown with the percentage of variance explained in parentheses. Different symbols represent individuals from different stations as indicated in the legend; sbb = Butimba Bay, sef = Trawl between Station E-F, sfg = Trawl between Station F-G, ste = Station E, stf = Station F, stg = Station G, sti = Station I, stj = Station J, stk = Station K. Note the separation of *E. antleter* between station E and station K on PC5 and PC6. Convex hulls are added for *E. antleter* to empasize differences between *E. antleter* from station E and station K.


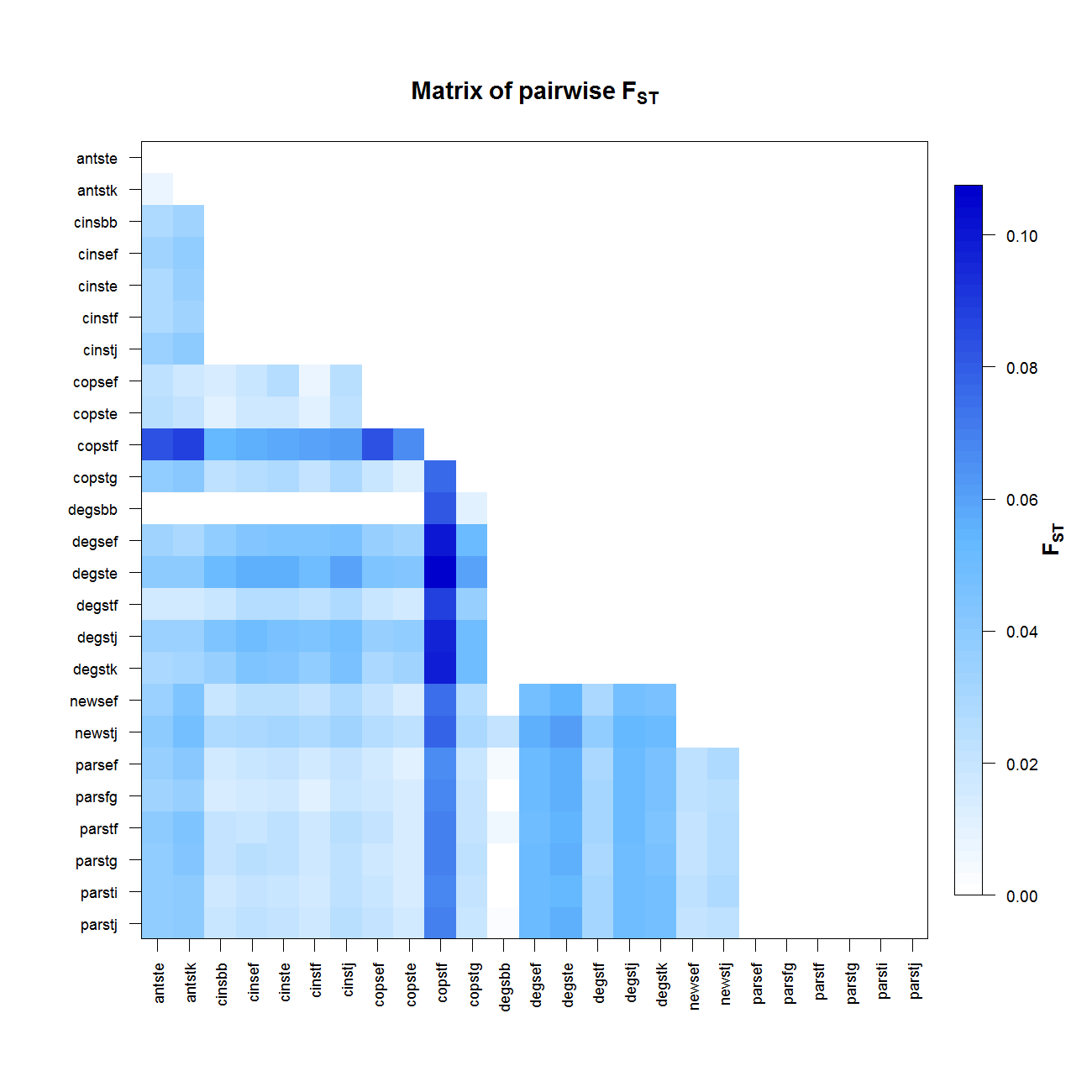


Figure S8. Pairwise divergence (F_ST_) between all eight studied species/populations at different stations. The F_ST_ values are based on a dataset of 8 609 biallelic SNPs with at least 1 minor allele count. The highest divergence is found between *E. coprologus “*Station F” and all other species/populations*.* None of F_ST_ values within species/populations between stations was significant except for the F_ST_ value of *E. antleter* between station E and K. Note that the low F_ST_ value between ”new degeni” from Butimba Bay and other species/populations is very low because of low sample size (n=1). Species abbrevations; ant = *E. antleter,* cin = *E. cinctus*, cop = *E. coprologus*, deg = ”new degeni”, new = *E.* “new invasive”, par = *E. paropius*. Station abbreviations; sbb = Butimba Bay, sef = Trawl between Station E-F, sfg = Trawl between Station F-G, ste = Station E, stf = Station F, stg = Station G, sti = Station I, stj = Station J, stk = Station K.
